# Some Old and New Theories of Calcification

**Published:** 1899-06

**Authors:** J. F. Bennett

**Affiliations:** London, England


### Abstracts and Translations.

### SOME OLD AND NEW THEORIES OF CALCIFICATION.[1]

BY MR. J. F. BENNETT, LONDON, ENGLAND.

THE paper of the evening was by Mr. J. F. Bennett, on "Some Old and New Theories of Calcification." Mr. Bennett passed in review the researches of George Rainey and of Professor Harting, of Utrecht, forty years ago, though the work of the latter did not become known in England until a much later date. Both those investigators believed they were able to reproduce artificially certain of the calcareous structures found in the animal kingdom, such as shells, spines, etc., and that their form in these tissues was largely due to the colloid solution in which they were deposited. The experiments leading to the enunciation of their views were detailed. The author dissented from the application of their theories to the teeth and bones, his objections being mainly based on the percentage composition of enamel, dentine, and bone,—viz., calcium phosphate 89.82 per cent., calcium carbonate 4.38 per cent., in one hundred parts of enamel. In one hundred parts of dentine the figures were: calcium phosphate 66.7 per cent., calcium carbonate

[1] Abstract of a paper read before the "Odontological Society of Great Britain," with discussion thereon.

3.36 per cent. Mr. Bennett proceeded to quote from one of Professor Harting's experiments, showing that phosphate of lime forming in a colloid in somewhat the same proportions as in bone, dentine, or enamel, could not assume the form of calcospherites. Harting's view was borne out by the experiments of Dr. Ord, particularly that depositing lime-salts in coagulated albumen at different temperatures in the proportions found in bone, when an even, continuous deposit was produced, without spheres. Mr. Bennett thought that Rainey's experiments with phosphates were disappointing, and particularized in what respects. The author thought experiment did not favor the globular form as a possible arrangement of the lime-salts in the teeth. He quoted from Tomes's great work on the proportions of the salts in bone and teeth, pointing out the discrepancies in the estimates of carbonates. He gave a digest of various opinions on calcospherites, in which idea there has been another "boom." But there remained the difficult question as to why calcospherites did not more often form in other albuminous constituents of the body,—*i.e.*, salivary glands, atheromatous patches, calcifying tubercle, etc. Mr. Bennett then dealt with Dr. Sims Woodhead's views on bone formation, as set forth in his paper before the Society in 1892. Dr. Woodhead showed there was always an increased amount of carbonic acid in the fluid near active cells, and phosphate and carbonate of lime were found, the latter small in quantity. Near dead membranes, if these salts were removed by dialysis they remained stable and might be deposited at once; but if kept in contact with the phosphoric acid of the blood and the alkaline phosphates they were redissolved.

The matrix might be looked upon as inert or dead organic matter, corresponding to the membrane through which dialysis occurred, such membrane (the matrix) serving to separate the lime-salts prepared in its neighborhood by the carbonic acid cells. The carbonic acid caused a throwing down of phosphate of lime, with a small proportion of lime, in which the phosphoric acid was generally replaced by carbonic acid. Professor Harting's union of lime-salts with albumen in calcoglobulin might have no connection with bone or dentine. The powerful influence of carbonic acid in determining calcification seemed to be more than merely holding the salts in solution and precipitating them on its removal. Mr. Bennett thought the union and molecular form of carbonate of lime which

Harting found in albumen might be attributable to some action of free carbonic acid evolved in the colloid solutions, causing a more intimate blending and modification of both organic and inorganic constituents. They had to learn not only how the lime-salts were deposited, but how they were prevented from depositing in other parts. Was carbonic acid the inhibitory influence? He thought Dr. Woodhead's views suggested a clearer idea of the cause of calcified structures in the ovary and other strange situations.

Mr. H. Lloyd Williams said the question which seemed to him extraordinary was that the basic substances of enamel and dentine respectively seemed totally different from each other. He asked Mr. Bennett whether carbonic acid had any influence in the matter. Apparently it did not greatly differ originally, because physiologically it could not be understood that there was any great difference between an odontoblast and an animal cell; yet the basic substance of enamel seemed to materially differ from that of dentine.

Mr. Storer Bennett said physiological views were altered about once every seven years, and so it was, apparently, with many of the teachings held a few years ago to be quite accurate. When he was a student the statement was not questioned that dentine was calcified by the deposition of calcospheroids, which fused into a solid substance, creating the matrix between the dentinal fibrils. Nobody seemed to have verified such a statement by actual observation. Even Mr. Tomes in his lectures years ago said the recognized view was so and so, but he did not say he had seen it, and quoted no observer who had. Some time ago he had urged that younger practitioners should conduct original investigations, and the older members of the profession might benefit by an attempt to disprove or verify the current text-book statements. The actual mode of formation of dentine would be an interesting field for investigation, which would reward the worker.

Mr. Ashley Barrett asked Mr. Bennett if he could say why the layer of dentine first calcified should assume that particular shape, —*i.e.*, globular spaces with convex boundaries. Why the granular layer should be as it was found was to him a difficult question.

The President asked Mr. Bennett his opinion as to the function of the cell, and the importance of the action of the cell in cases of calcification. He presumed a special cell was engaged in the production of dentine; also a special cell was engaged in the calcification of enamel. He questioned whether there were any cells connected with the calcification which occurred in the structure of the pulp as met with.

Mr. Brunton said he was not familiar with the question, but probably a little light might be thrown upon the deposition of lime by observing the fresh-water snail, for if the body of one were taken out of its shell and dropped into water, it would be found, in a few hours, that this fresh-water snail had built itself a new house.

Mr. J. F. Bennett, in reply, thought his paper had fallen on unfortunate times as regards discussion. He invited members to read his paper. When a man like Dr. Woodhead came to the meeting, he sometimes propounded a new theory which they had been in the habit of taking as gospel. Then a new theory was advanced, was perhaps not much noticed, and they found themselves holding views the basis of which they had never troubled to properly examine. Every one would respect Mr. Tomes, who was a master of the subjects he wrote and talked on. Mr. Tomes had gone into the question as thoroughly as Rainey, and if he found grounds to believe in the possibility of Rainey's theory in any form, well and good. Mr. Bennett invited the members, especially the junior ones, to read a few of the treatises, for investigation might result. The functions of the cells, of course, might be considered from two points of view. They might have a function like the odontoblast, or the mesoblast, or the osteoblasts, or in forming the matrix. The animal portion of the structure also has a part to play in the laying down of the cells as in the mesoblast, certainly in the osteoblast, and probably in the odontoblast. A special action of the cell which Dr. Woodhead dwelt upon was the new idea that it was a carrier of carbonic acid. It had long been known that carbonic acid held in solution the phosphates of lime circulating in the blood, and by passing off in vapors, or in some other way, the lime-salts were precipitated. It was well known that saliva contained phosphate of lime which had been in the mouth in soluble form, but as soon as the carbonic acid passed in, it was precipitated in the form of tartar on the teeth. Dr. Woodhead suggested that the action of carbonic acid was far more difficult: it was continually holding and releasing the phosphate of lime, performing that process repeatedly in the blood. But its special function was to hold the carbonic acid and retain command over the lime-salts, either to make them soluble or the reverse. Therefore, when the connective tissue corpuscles or leucocytes were round a tuberculous nodule in vast quantities, in order to isolate the bacteria, the cells carried carbonic acid to some which were really dead in front of them, and so deposited the lime. Mr. Barrett had spoken of the margin of early formed dentine. He did not quite know whether he meant the convexities which were found at the very margin, something like a series of waves. That had been explained by some as representing the little points where the enamel prisms fixed against the dentine, but that was not a very adequate explanation. The granular layer was supposed to be a mere broken and interglobular dentine. The point he wished to bring out in his paper was that, although they were very eager to find out what interglobular dentine was,—which was unquestionably the key to the structure of dentine,—they were not obliged to take Rainey's theory. If they thought it was due to calcospheroids, it was curious that nobody had been able to evolve a calcospheroid out of a composition of dentine and bone, and that it was only a product which could be formed by carbonate of lime—not phosphate. Mr. Brunton had alluded to the fresh-water snail and its new mantle secreting in a short space of time. This was the case with the lobster and many other animals. He did not dispute that Rainey was wrong regarding gastropods and that kind of thing, because it was known that the shell was formed of carbonate of lime. Artificial spheroids could be formed in carbonate of lime. The difficulty occurred where phosphate of lime had to be dealt with.—*Journal of the British Dental Association.*

